# Neurobiology and Cognition in Girls at High‐Risk of Eating Disorders: Exploring Imaging‐Derived Trait Markers

**DOI:** 10.1002/erv.3203

**Published:** 2025-04-24

**Authors:** E. Pappaianni, B. Borsarini, C. Berchio, S. Aicoboaie, S. V. Konstantopoulou, D. Van de ville, N. Micali

**Affiliations:** ^1^ Center for Eating and Feeding Disorders Research (CEDaR) Mental Health Center Ballerup Copenhagen University Hospital—Mental Health Services CPH Copenhagen Denmark; ^2^ Network Plasticity Modulation (NetPM) Lab Department of Clinical Neurosciences Faculty of Medicine University of Geneva Geneva Switzerland; ^3^ Department of Translational Biomedicine and Neuroscience Group of Psychiatric Neuroscience University of Bari Bari Italy; ^4^ Institute of Biological Psychiatry Mental Health Centre Sct Hans Copenhagen University Hospital ‐ Mental Health Services Copenhagen Denmark; ^5^ Network Plasticity Modulation (NetPM) Lab Department of Clinical Neurosciences, Faculty of Medicine, University of Geneva Geneva Switzerland; ^6^ Neuro‐X Institute École Polytechnique Fédérale de Lausanne Geneva Switzerland; ^7^ Department of Radiology and Medical Informatics University of Geneva Geneva Switzerland; ^8^ Great Ormond Street Institute of Child Health University College London London UK

**Keywords:** brain morphometry, eating disorders, high‐risk offspring, neurocognition, trait markers

## Abstract

**Background:**

Eating disorders (EDs) are serious psychiatric disorders characterized by impairments in neurocognition and altered brain structure. To date the majority of studies have investigated these in acutely ill or recovered individuals. Studying children at familial high risk (FHR) for psychiatric disorders allows investigating vulnerability traits or trait markers that may be present before disorder onset. Our study is the first one to examine executive function and brain structure in girls at FHR for ED (Anorexia Nervosa, Bulimia Nervosa, and Binge Eating Disorder) compared to controls (girls not at familial high risk ‐ HC).

**Methods:**

Forty‐six (46) FHR girls (median age: 10.5 years, range: 9) and 50 HC girls (median age: 12 years, range: 8) completed a battery of neuropsychological tests assessing cognitive flexibility, inhibitory control, and working memory. Structural magnetic resonance imaging assessed grey matter volume (GMV) and cortical thickness (CT).

**Results:**

Girls at FHR for ED performed a higher number of errors in a cognitive flexibility task compared to HC (*β* = 0.15, *p* < 0.05). They also had increased GMV in posterior regions such as the right supramarginal gyrus, middle occipital gyrus, and lingual/fusiform gyrus compared to HC (*p* < 0.05 cluster‐level FWE‐corrected), as well as increased CT in the left transverse pole (*p* < 0.001) and right posterior cingulate cortex (*p* < 0.05).

**Conclusions:**

Girls at FHR show characteristic neurocognitive performance similar to that seen in individuals with ED, as well as differences in brain structure compared to HC. Our findings, together with previous evidence, highlight impairment in cognitive flexibility as a possible trait marker of ED. Further longitudinal studies are needed to confirm differences in GMV and CT identified in this study.

**Summary**

- This is the first study to examine executive function and brain structure in healthy girls at familial high risk (FHR) for eating disorders (ED) compared to controls.
- Girls at FHR for ED performed worse in a cognitive flexibility task compared to controls and showed differences in grey matter volume and cortical thickness in specific brain regions.
- Our findings highlight impairment in cognitive flexibility as a possible trait marker of ED, with further longitudinal studies needed to confirm the differences in grey matter volume and cortical thickness identified in this study.

## 1 Introduction and Background

Eating disorders (EDs), including anorexia nervosa (AN), bulimia nervosa (BN) and binge eating disorder (BED), are serious psychiatric illnesses characterised by dysfunctional eating behaviour (Treasure et al. 2020). Behaviours associated with EDs include restrictive eating or avoidance of specific foods, binge eating, and purging (e.g., self‐induced vomiting, laxative misuse, or compulsive exercise). Although each of these disorders is associated with distinct clinical features, they affect individuals overall health, at a physical, psychological, and social level (Treasure et al. 2020). EDs pose a significant threat to well‐being and are among the most dangerous mental health conditions (Chesney et al. 2014). Epidemiological data show that the prevalence of EDs has been increasing over the past decade, resulting in an estimated 7.8% of the world's population suffering from EDs (Galmiche et al. 2019).

In addition to clinical symptoms, a large body of literature shows EDs are associated with deficits in executive function (Diaz‐Marsa et al. 2023), that is, a compendium of top‐down regulatory processes used in situations where recourse to automatic responses or reliance on instinct and intuition would be inadvisable and not feasible (Diamond 2013). Cognitive flexibility (Diaz‐Marsa et al. 2023), inhibitory control (Wu et al. 2013; Bartholdy et al. 2016), and working memory (Brooks et al. 2017; Dahlén et al. 2022) appear to be the most affected aspects of executive function in EDs. Cognitive flexibility, also known as ‘set shifting’, is defined as the ability to shift between tasks, approaches and mental sets (Diamond 2013); inhibitory control refers to the ability to exert control over attention, actions, thoughts and/or emotions (Diamond 2013), while working memory refers to the ability of holding and manipulating information in mind (Diamond 2013). Executive functions vary across EDs; for example, individuals with AN present with extreme behavioural rigidity and lack of flexibility (Miles, Gnatt, Phillipou, and Nedeljkovic 2020a), just as impulsivity and lack of inhibitory control (such as during a binge episode) are present in individuals with BN and especially BED (Boswell and Grilo 2021).

In parallel to neurocognitive characteristics, specific brain morphometric features (e.g., grey matter volume (GMV) or cortical thickness (CT) characteristics) have been observed in individuals with EDs. Although it is well recognized that EDs have a neurobiological component (Bulik et al. 2022), the evidence about brain structural characteristics being state versus trait markers of EDs is mixed. In particular, studies of acutely ill (often underweight) and recovered individuals with AN show GMV reduction in subcortical regions such as the thalamus, and global and focal CT reduction in parieto‐temporal areas (Walton et al. 2022). These differences seem to persist, although at lower severity, in partially weight‐restored individuals (Walton et al. 2022). For what concerns individuals with BN and BED, increased GMV has been reported in frontal and ventral striatal areas (Van den Eynde et al. 2012), fusiform gyrus and insula (Chen et al. 2022), and nucleus accumbens (Abdo et al. 2020). To our knowledge, no studies have investigated brain structure of recovered individuals with BN and BED.

Studying populations at high risk for a psychiatric disorder is one way to study whether state versus trait markers of disease (particularly in the brain) might be ‘scars’ of the disorder. Familial high risk (FHR) status in particular has been applied to the study of mental health disorders, such as EDs, that are heritable (Bulik et al. 2019). Evidence for family aggregation in AN is strong (Steinhausen et al. 2015), and heritability estimates are reported to be 50%–60% in AN (Yilmaz et al. 2015; de Jorge Martínez et al. 2022), 41% in BN (Yao et al. 2021), and 41%–57% in BED (Munn‐Chernoff and Baker 2016; Donato et al. 2022). BN and BED also aggregate in families and are heritable (Bulik et al. 2019). The FHR design has been used extensively across other clinical populations (Thorup et al. 2015; Andreou et al. 2023; Talati et al. 2013; Tierney et al. 2012; Luyster et al. 2011) including schizophrenia, bipolar disorder, depression and autism, but very little yet with EDs (Kothari et al. 2013, 2014). In our preliminary FHR study (Pappaianni et al. 2023) we showed that, compared to healthy controls, healthy girls at FHR for EDs (i.e., daughters of mothers with lifetime history of ED) reported differences in executive function specifically in cognitive flexibility and in patterns of functional connectivity during resting‐state functional Magnetic Resonance Imaging (MRI). This preliminary evidence supports the hypothesis that specific neurocognitive characteristics may be trait markers of EDs, but these findings need to be replicated in a larger sample. In addition, our previous study only investigated functional brain characteristics, not brain‐structure measures (such as GMV and CT) in children at FHR for EDs. Therefore, this study wished to assess children at FHR for EDs across neurocognition and brain structure. In the current study we aimed to extend our previous study (Pappaianni et al. 2023) by including healthy girls at FHR for a broader range of EDs (AN, BN and BED) and controls, and to compare executive function, specifically the domains of cognitive flexibility, inhibitory control, and working memory; as well as structural morphology across these two groups. In light of our previous studies (Kothari et al. 2013, 2014; Pappaianni et al. 2023), we hypothesised that girls at FHR for EDs would perform worse, especially in cognitive flexibility, than control girls. Given differences expected across EDs, we aimed to explore executive function across specific EDs (AN, BN and BED). From a neural point of view, this is the first study to investigate the brain structure in girls at FHR for EDs. Therefore, we expected to find differences in GMV and CT between the two groups that would mirror those found in individuals with active EDs, that is in subcortical regions and in frontal, temporal and parietal areas.

## 2 Methods and Materials

### 2.1 Participants

This study was run in Switzerland (Geneva Canton, between 2019–2023, given the COVID‐19 pandemic the study lasted longer than initially planned). Mothers and daughters were recruited from ED treatment centres, private clinics, and the general population in the Geneva and the adjacent Vaud Canton. To be included in the ED‐FHR group, mothers had to have a current or lifetime diagnosis of ED (AN, BN or BED) according to DSM‐5 diagnostic criteria (Diagnostic and Statistical Manual of, 2013). For inclusion in the HC group, mothers had to report no current or past psychiatric disorder (including ED). Daughters were eligible for the study if they were between the ages of 8 and 15 years old. Exclusion criteria for all daughters included the presence of either ED symptoms with impact or subthreshold/full‐blown ED, a history of neurological disease, and intake of medication affecting the central nervous system. Girls who were receiving any pharmacological treatment were not included in the study. We did not prevent siblings from participating in the study if they met the inclusion criteria. A recruitment flowchart is provided in Supporting Information S1.

### 2.2 Measures

#### 2.2.1 Socio Demographic Data

Maternal age, ethnicity, marital status, educational attainment, as well as daughters' age and ethnicity were collected via an ad hoc questionnaire for mothers.

#### 2.2.2 Psychopathology

##### 2.2.2.1 Mothers

Initial screening for maternal psychiatric disorders for HC mothers was performed using the Structural Clinical Interview for DSM‐5 Research Version (SCID‐5‐RV) (American Psychiatric Association, 2015) checklist. Maternal diagnoses of ED were ascertained using the ED module of the SCID‐5 RV (American Psychiatric Association, 2015). This module incorporates open‐ended inquiries regarding the manifestation of ED symptoms and associated cognitions. The SCID‐5 (American Psychiatric Association, 2015) ED module was administered by a trained research psychologist (BB) and all diagnoses were conferred together with the project leader, a psychiatrist with expertise in EDs (NM). Women with EDs were given a lifetime diagnosis (and a current diagnosis if they had an active ED at interview).

##### 2.2.2.2 Offspring

We assessed girls' psychopathology using the Development and Wellbeing Assessment (DAWBA), a widely used semi‐structured tool for detecting mental health problems and psychiatric diagnoses in young people (Goodman et al. 2000). The DAWBA was completed by mothers (for those < the age of 11 years), while daughters were additionally asked to complete the measure if they were ≥ 11 years of age (as per DAWBA instructions). The DAWBA (Goodman et al. 2000) uses a computer algorithm to generate probabilities of psychiatric diagnoses according to the criteria outlined in the fifth edition of the Diagnostic and Statistical Manual of Mental Disorders (DSM‐5) (American Psychiatric Association, 2013). The generated probabilities of disorder are then rated by a trained clinical rater. The DAWBA was completed prior to other assessments and girls who presented exclusion criteria were excluded from participation.

#### 2.2.3 Anthropometry and Pubertal Stage

Weight and height were measured by a trained research assistant in order to calculate BMI centiles and Weight‐for‐Height. Mothers were asked to rate their daughters' pubertal and breast development using Tanner's Stages (Marshall and Tanner 1969), a pictogram containing scaled drawings illustrating external sexual characteristics such as breast and pubic hair development.

#### 2.2.4 Intelligence

Four subtests of the Wechsler Intelligence Scale for Children‐Fifth Edition (WISC‐V) (Wechsler 2014) were administered as a measure of general intelligence. Vocabulary test (VC), Similarity Test (SI), Block Design (BD) and Matrix Reasoning (MR). A proxy for the Verbal Comprehension Index (VCI) was obtained by summing VC and SI scores. A proxy for the Perceptual Reasoning Index (PRI) was also obtained by summing BD and MR.

### 2.3 Neurocognitive Testing

Neurocognitive function was assessed using tasks from the Cambridge Neuropsychological Test Automated Battery (CANTAB, Cambridge Cognition, https://cambridgecognition.com/digital‐cognitive‐assessments/). Audio instructions in French for each task were provided by the CANTAB app, followed by task‐specific training. The following subtests were included in our study‐specific battery: the Multitasking Test (MTT) for cognitive flexibility, the Stop Signal Task (SST) for inhibitory control, and the Spatial Working Memory Task (SWM) for working memory.

#### 2.3.1 Multitasking Test

The MTT assesses the ability to switch attention effectively between arrow positions and directions, ignoring irrelevant information in the midst of potential distractions. Arrows are displayed on either side of the screen, pointing either left or right. A cue at the top of the screen instructs participants to press left or right depending on the direction or position of the arrow. Some trials are consistent with both position and direction (e.g., the arrow on the right‐side points to the right), while others are inconsistent and require more cognitive effort. We measured performance indicators such as total number of incorrect responses (across all trials), the median reaction latency, the incongruency cost (i.e., the difference between the median latency of response in congruent trials vs. incongruent trials), and the multitasking cost (i.e., the difference between the median latency of response during assessed blocks in which both rules are used vs. blocks in which only a rule is used).

#### 2.3.2 Stop Signal Task (SST)

The SST represents a distinctive variation of a traditional assessment of response inhibition or impulse control. In this task, participants are required to react to an arrow stimulus by choosing between two options based on the arrow's direction. When accompanied by an audible tone, participants must exercise restraint in their response. The task allows adaptation to the participant's performance and a focus on achieving a 50% success rate for inhibition. Key outcome measures encompass reaction times (i.e., the estimate of time where an individual can successfully inhibit their response 50% of time), total number of missed trials, directions errors during a “Go” trials and directions errors during “Stop” trials.

#### 2.3.3 Spatial Working Memory (SWM)

The SWM assesses the retention and manipulation of visuospatial information. This self‐administered test places significant demands on executive function, offering insights into working memory performance and the use of strategies to complete the task. The test commences with a series of coloured squares (boxes) presented on the screen. The goal is for the participant, through the selection of boxes and a process of elimination, to locate a yellow ‘token’ in various boxes and use it to fill an empty column on the right side of the screen. Depending on the chosen difficulty level, the number of boxes can incrementally increase, reaching a maximum of 12 boxes. Outcome measures encompass number of total errors, a measure of strategy for 6–8 boxes or 6–12 boxes (i.e., total number of times a subject begins a new search pattern from the same box they started with previously), and between errors measures (i.e., the number of times the subject revisits a box in which a token has previously been found) related to 4, 6, 8, 12 boxes, as well as a general index of between errors calculated across all assessed 4, 6, 8 token trials.

### 2.4 Experimental Procedure

#### 2.4.1 Baseline Visit

An initial visit was held at the Service de Psychiatrie de l’Enfant et l’Adolescent (SPEA), at the Hôpitaux Universitaires de Genève (HUG), Geneva (Switzerland). After providing informed consent, mothers completed all measures. Daughters underwent anthropometric assessment and completed the neurocognitive battery and intelligence evaluation.

#### 2.4.2 MRI Visit

The MRI session was held at the Foundation Campus Biotech Geneva (FCBG), Geneva (Switzerland). Girls participated in a short mock MRI session to familiarise themselves with the scanning procedure. Girls at FHR for EDs and controls were then scanned on a Siemens MAGNETOM Prisma 3‐Tesla MRI research scanner. A structural T1‐weighted Magnetization Prepared Rapid Acquisition Gradient Echo (MPRAGE) image was acquired. All MRI acquisition parameters and procedure are described in Supporting Information S1.

### 2.5 Ethical Approval

Ethical approval for the study (ID 2018–02363) was granted by the Swiss Association of Research Ethics Committees “SwissEthics” (https://swissethics.ch/en). Both mother and daughter (only if older than 14 years) were provided informed consent for participation. The younger girls were asked to give verbal consent to participate in the study after being introduced to the experimental procedures using age‐appropriate language.

### 2.6 Data Analysis

#### 2.6.1 Demographic Data, Psychopathology, and Neurocognitive Function

Each variable was examined individually to identify inconsistencies or outliers and to assess its distribution. Demographic variables and covariates were compared between groups using chi square or Fisher's exact tests for categorical variables and Mann‐Whitney *U* test for continuous variables. Dichotomous variables were created for breast development using Tanner Stages (Prepubertal and pubertal vs. Postpubertal) and for the presence/absence of psychiatric diagnoses.

Neurocognitive functions were analysed using unadjusted linear regression with each CANTAB outcome set as the dependent variable and following adjustment for age, VCI, and presence of psychiatric diagnosis as independent variables. Bootstrapped estimation of coefficients (5000 replicates) was used to confirm the accuracy of sample estimates. The Bonferroni correction for multiple comparisons was applied to significant results by adjusting the significance threshold according to the number of outcome variables within each neurocognitive task.

An exploratory analysis using the same method was applied to examine neurocognitive functions across the different subgroups of maternal EDs (AN, BN, BED), focussing only on significant results from the previous analysis.

Variables were subjected to logarithmic or *Z* score transformation to comply with the assumption of normality. All statistical analyses were carried out using SPSS v.28 (SPSS Statistics Editions · IBM SPSS) and JASP v. 0.18.2 (https://jasp‐stats.org/). Statistical significance threshold was set at *p* ≤ 0.05 for all analyses.

#### 2.6.2 MRI Analysis

##### 2.6.2.1 Voxel‐Based Morphometry

The Computational Anatomy Toolbox v. 12 (CAT12, https://neuro‐jena.github.io/cat/) as an extension of Statistical Parametric Mapping v. 12 (SPM12) toolbox (http://www.fil.ion.ucl.ac.uk/spm/), working in MATLAB (TheMathworks), were used for MRI analysis. Voxel‐based morphometry (Ashburner and Friston 2000) (VBM) as univariate morphometric technique was used to investigate differences in local brain anatomy between FHR children and controls. VBM examines GM concentration at the voxel level and investigates differences between groups. Detailed description of VBM analysis is available in Supporting Information S1.

Statistical analysis was based on a mass univariate approach using general linear models (GLMs). A second‐level GLM matrix (two‐sample *t*‐test) was designed and estimated, including age, TIV and presence of psychiatric comorbidities as covariates of interest. A threshold masking of 0.2 was chosen to restrict the results to brain areas in the analysis. Two contrasts of interest were defined: FHR > HC control, to detect voxels with higher GM in FHR children than in controls; and HC > FHR, to detect possible voxels with higher GM in HC children than in FHR children. Statistical significance level was set at *p* < 0.05 Family‐Wise Error (FWE)‐corrected at the cluster level.

##### 2.6.2.2 Regions‐Of‐Interest Analysis—Cortical Thickness

CT measurements were extracted automatically with CAT12, that uses a projection‐based thickness approach to handle partial volume information, sulcal blurring and sulcal asymmetry without explicit sulcal reconstruction (Dahnke et al. 2013). The Desikan‐Killiany (DK40) Atlas (Desikan et al. 2006) was used as a reference to extract CT information from each of the 68 regions of interest (ROIs) (34 cortical ROIs per hemisphere). ROI‐specific CT measurement entered different linear regression analyses (one per ROI), where CT value was dependent variable, group, age presence of comorbidities and TIV as independent variables. Analyses were run using JASP v. 0.18.2 (https://jasp‐stats.org/). Statistical significance threshold was set at *p* ≤ 0.05.

## 3 Results

Forty‐six (46) girls at FHR (median age: 10.5 years, range: 9) and 50 HC girls (median age: 12 years, range: 8) were included in the study. At the time of evaluation, no mother or girl reported any discrepancy between biological sex and gender. Full details of the recruitment process are available in Supporting Information S1: Figures S1 and S2.

### 3.1 Demographic Data

Groups differed in age (*χ* ^2^ = 5.19, *p* = 0.02), but not in breast development, Weight for Height, VCI, or PRI (*p* > 0.05). Number of siblings included in the study did not differ between girls at FHR of ED and controls (*N* = 6 per group, i.e., 3 pairs of siblings per group, *p* > 0.05).

Mothers differed in age between groups (*χ* ^2^ = 43.38, *p* = 0.006), but they were comparable for education, ethnicity, and marital status (all *p* > 0.05).

All demographic details of girls and mothers, as well as relative comparisons, are available in Table 1.

**TABLE 1:** Demographic characteristics of girls at FHR for EDs and HC and their mothers.

	Group		Statistics
	FHR *N* = 46	HC *N* = 50	
Maternal characteristics			
Age (years), Median (range)	42 (21)	45.5 (21)	*χ* ^2^ = 43.38, *p* = 0.006
Eating disorder (*n*)	AN (17) BED (17) BN (12)		
Remission (*n*)	No remission (19) Partial remission (11) Full remission (16)		
Education, *N* (%)			
University or higher	31 (32.29%)	39 (40.63%)	Fisher's exact test = 0.53, *p* = 0.26
Up to A levels	15 (15.63%)	11 (11.46%)	
Ethnicity, *N* (%)			
White European Other (eg., African)	40 (41.67%)	43 (44.79%)	Fisher's exact test = 0.08, *p* = 1
	6 (6.25%)	7 (7.39%)	
Marital status, *N* (%)			
Married Single Other	29 (30.21%)	39 (40.63%)	
			*χ* ^2^ = 3.05, *p* = 0.22
	2 (2.08%)	3 (3.13%)	
	14 (14.58%)	8 (8.33%)	
Offspring characteristics			
Age (years), Median (range)	10.5 (9)	12 (8)	*χ* ^2^ = 5.19, *p* = 0.02
BMI centile, median (range)	64.67 (96.49)	37.76 (99.31)	*U* = 1381.50, *p* = 0.09
Weight for height, median (range)	105.15 (59.11)	96.26 (16.47)	*U* = 1383, *p* = 0.09
Tanner stages (brest development)—N (%)			
Prepubertal and pubertal	35 (37.23%)	28 (29.79%)	Log OR = 0.81, *p* = 0.08
Postpubertal	11 (11.70%)	20 (21.28%)	
Tanner stages (pubic development)—N (%)			
Prepubertal and pubertal	34 (35.42%)	24 (25%)	Log OR = 1.11, *p* = 0.01
Postpubertal	12 (12.50%)	26 (27.08%)	
WISC‐V			
Median (range)			
Verbal comprehension index (VCI)	26 (18)	25 (20)	*U* = 1093, *p* = 0.80
Perceptual reasoning index (PRI)	23 (20)	23 (20)	*U* = 1057, *p* = 0.60
Presence of a psychiatric disorder (DAWBA)—N (%)	19 (20%)	6 (6.31%)	Log OR = −1.60, *p* = 0.002

*Note:* Statistics reported group comparisons based on Chi‐square (χ^2^), Fisher's exact test (OR = Odd Ratio) or Mann‐Whitney (U) tests depending on variables distribution.

#### 3.1.1 Psychopathology

##### 3.1.1.1 Mothers

Seventeen (17) mothers had a lifetime diagnosis of AN, 17 of BED, and 12 mothers of BN. Among mothers with AN, the average duration of illness was 16.6 years (standard deviation (SD) = 8.9). For mothers with BED, the mean duration of illness was 18.5 years (SD = 8.0). Finally, mothers diagnosed with BN had a mean duration of illness of 14.0 years (SD = 8.8).

##### 3.1.1.2 Offspring

Girls at FHR of ED had a higher prevalence of psychiatric diagnoses (*χ* ^2^ = 10.264, *p* = 0.001) compared to control girls (see Table 1). According to the DAWBA assessment, nearly one‐third (33%) of girls at FHR for ED had an emotional disorder, while 11% exhibited a behavioural disorder.

### 3.2 Neurocognitive Function

All neurocognitive function results are presented in Table 2. Girls at FHR for EDs showed a worse performance (*β* = 0.15, *p* < 0.05) in MTT, that is higher number of total number of incorrect responses adjusting for age, VCI, and presence of psychiatric diagnoses (F_(4,94)_ = 20.50, *p* < 0.001, *R* ^2^ = 0.47). This result was confirmed by bootstrapping based on 5000 replicates.

**TABLE 2:** Neurocognitive characteristics in girls at FHR for ED and HC: results from unadjusted and adjusted linear regression models.

	*Unadjusted analysis*	*Adjusted* *^a^* *analysis*
MTT—ICMD (incongruency cost)	*β* = –*0.18, F*_*(1,95)*_ *= 1.46*	*β* = –*0.12, F*_*(4,94)*_ *= 0.47*
MTT—LMD (median reaction latency)	*β* = *0.04, F*_*(1,95)*_ *= 5.01*	*β* = *0.01, F*_*(4,94)*_ *= 5.25*
MTT—MTCMD (median Multitasking cost)	*β* = –*0.10, F*_*(1,95)*_ *= 0.99*	*β* = *0.03, F*_*(4,94)*_ *= 2.04*
MTT—TIC (total incorrect)	*β* = *0.90* *^b^* *, F*_*(1,95)*_ *= 14.13*	*β* = *0.15* *^c^* *, F*_*(4,94)*_ *= 20.50*
SST—DEG (direction errors: Go trials)	*β* = *0.07, F*_*(1,95)*_ *= 0.41*	*β* = –*0.12, F*_*(4,94)*_ *= 9.54*
SST—DES (direction errors: Stop trials)	*β* = –*0.05, F*_*(1,95)*_ *= 2.04*	*β* = –*0 to* –*52, F*_*(4,94)*_ *= 2.51*
SST—MT (Missed trials)	*β* = *0.20* *^c^* *, F*_*(1,95)*_ *= 5.52*	*β* = –*0 to* –*12, F*_*(4,94)*_ *= 14.67*
SST—SSRT (stop signal reaction time)	*β* = –*0.02, F*_*(1,95)*_ *= 0.18*	*β* = –*0.06, F*_*(4,94)*_ *= 3.18*
SWM—BE12 (between errors 12 boxes)	*β* = *0.12, F*_*(1,95)*_ *= 3.85*	*β* = *0.04, F*_*(4,94)*_ *= 4.28*
SWM—BE4 (between errors 4 boxes)	*β* = –*0.01, F*_*(1,95)*_ *= 0.04*	*β* = –*0.04, F*_*(4,94)*_ *= 2.19*
SWM—BE468 (between errors across 4, 6, 8 boxes)	*β* = *0.08, F*_*(1,95)*_ *= 0.66*	*β* = –*0.09, F*_*(4,94)*_ *= 4.56*
SWM—BE6 (between errors 6 boxes)	*β* = *0.01, F*_*(1,95)*_ *= 0.03*	*β* = –*0.11, F*_*(4,94)*_ *= 3.10*
SWM—BE8 (between errors)	*β* = *0.10, F*_*(1,95)*_ *= 1.18*	*β* = –*0.07, F*_*(4,94)*_ *= 4.94*
SWM—S (strategy 6‐8 boxes)	*β* = *0.02, F*_*(1,95)*_ *= 0.26*	*β* = –*0.019, F*_*(4,94)*_ *= 1.34*
SWM—SX (strategy 6‐12 boxes)	*β* = *0.02, F*_*(1,95)*_ *= 0.39*	*β* = –*0.02, F*_*(4,94)*_ *= 2.36*
SWM—TE468 (total errors across 4, 6 and 8 boxes)	*β* = *0.08, F*_*(1,95)*_ *= 0.70*	*β* = –*0.08, F*_*(4,94)*_ *= 4.50*

^a^ Adjusted for age, Verbal Comprehension Index (VIC) and presence of psychiatric diagnoses.

^c^ *p* < 0.05.

^b^ *p* < 0.001.

Given the significant effect of age (*p* < 0.001) and the presence of outliers, for these analyses we excluded participants aged ≥ 15 years in both groups (*N* = 2 for FHR, *N* = 11 for HC). The new subsample consisted of 44 FHR and 39 HC, with a median age of 10 in FHR and 11 in HC (range = 6, min = 8, max = 14). Age did not differ between groups (*X* ^2^ = 4.46, *p* = 0.61). We repeated all neurocognitive analyses using CANTAB scores as the dependent variable, group as a factor, and adjusting for VIQ and presence of comorbidities. Bootstrap estimation of the coefficients (5000 replicates) was used to confirm the accuracy of the sample estimates. The results confirmed that girls at FHR for EDs exhibited poorer performance in MTT (*β* = 0.22, *p* = 0.002). This finding remained significant after applying the Bonferroni correction to the MTT analyses, which involved separate linear regressions for each outcome variable (adjusted significance threshold: *p* = 0.012; 0.05/4).

No other statistically significant differences were observed in MTT, SST, or SWM.

#### 3.2.1 Exploratory Stratified Analysis

Exploratory analyses compared neurocognitive function in girls at FHR for each ED (AN, BN, and BED) versus HC. Neurocognitive outcomes were compared between groups, adjusting for age, VCI and presence of psychiatric diagnoses. Unadjusted analyses revealed a significant effect of group in total number of incorrect responses on the MTT (F_(3,95)_ = 5.25, *p* = 0.002); that is girls at FHR for AN had a worse performance compared to HC (*β* = 0.38, *p* < 0.001), as did girls at FHR for BED (*β* = 0.27, *p* = 0.02), while only a trend towards statistical significance emerged for girls at FHR for BN versus HC (*β* = 0.22, *p* = 0.09). Findings did not remain statistically significant when adjusting for covariates. No other statistically significant differences emerged between groups in MTT, SST, and SWM.

### 3.3 MRI Analysis

#### 3.3.1 Voxel‐Based Morphometry

Eight girls (4 girls at FHR and 4 HC) did not participate in the MRI session, leaving a samples size of 42 for girls at FHR and 46 HC.

Groups did not differ in terms of GM, WM and CSF volumes, as well as TIV (*p* > 0.05).

VBM returned 3 significant clusters of voxels between groups, specifically related to the contrast FHR > HC (i.e., higher GM volume in FHR than HC). For visualisation purposes, *p* < 0.001 (uncorrected) was set, with extent threshold *k* = 650. The three clusters were all located in posterior areas of the right hemisphere. The first cluster (*k* = 1641, P_FWE‐corr_ = 0.002 at cluster‐level) was located in the right postcentral/supramarginal gyrus; the second cluster (*k* = 1496, P_FWE‐corr_ = 0.002 at cluster‐level) in the right middle occipital gyrus; the last cluster (*k* = 1031, P_FWE‐corr_ = 0.016 at cluster‐level) included areas such as the right lingual gyrus. See Table 3 for all details about VBM. No statistically significant differences were found in the opposite contrast (HC > FHR). VBM was corrected for age, TIV and presence of psychiatric diagnoses.

**TABLE 3:** GMV results. Area label, size (*k*) of the cluster, spatial coordinates and significance level related to significant different GMV between offspring at FHR of ED and controls. Contrast FHR > HC.

*Area*	*K voxels*	*Significance (P_FWE‐corr_ at cluster‐level)*	*Spatial coordinates*
Right supramarginal gyrus/postcentral gyrus	1641	0.002	59–8–54
Right middle occipital gyrus	1496	0.002	53–69–24
Right lingual gyrus/fusiform gyrus	1031	0.016	24–47–3

#### 3.3.2 Cortical Thickness—ROIs Analysis

**FIGURE 1:**
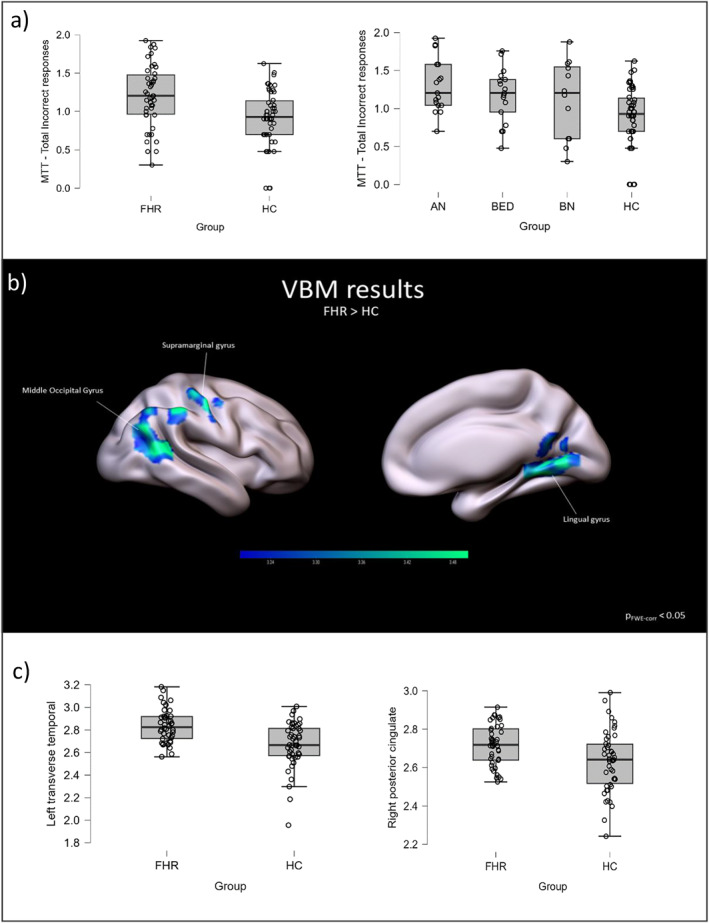
Panel (a) On the left, boxplot illustrating the distribution of total incorrect responses (log transformed) between girls at FHR for EDs and HC. On the right, boxplot illustrating the same distribution split based on maternal diagnosis. Panel (b) VBM results. GM volume in three different clusters showing statistically significant differences between girls at FHR and controls. Panel (c) boxplots illustrating the distribution of CT in two significant ROIs (left transvers temporal gyrus and right posterior cingulate gyrus) between groups.

Global CT did not differ between groups (*p* > 0.05). ROI analysis revealed two regions with significantly different CT between groups: the left transverse temporal gyrus (DK40's label: *ltransversetemporal*) and the right posterior cingulate gyrus (DK's label: *rposteriorcingulate*). The direction of the results is consistent: girls at FHR for EDs showed higher CT in both the left transverse temporal gyrus (F(4,87) = 5.472, *p* < 0.001, *R* ^2^ = 0.209, effect of group ≤ 0.001) and the right posterior cingulate gyrus (F(4,87) = 4.501, *p* = 0.002, *R* ^2^ = 0.178, effect of group = 0.031). Analyses were adjusted for age, TIV, and presence of psychiatric diagnosis. Both results were confirmed by bootstrapping based on 5000 replicates (*p* = 0.039 and *p* ≤ 0.001 respectively for group factor). Distribution of variables and group comparisons are available in Figure 1c. No other statistically significant ROIs emerged between groups for CT adjusting for covariates of interest.

## 4 Discussion

In this FHR study we investigated neurocognitive function and brain structure in girls at FHR for EDs compared to control girls. To our knowledge, this is the first study to combine behavioural measures (executive function) and structural brain imaging (with a focus on GM) in an FHR sample.

Girls at FHR for EDs had worse performance (i.e., they generally made more mistakes) than control girls on a cognitive flexibility task. This difference confirms our hypothesis and is in line with evidence from our preliminary study (Pappaianni et al. 2023), in which girls at FHR of ED showed worse cognitive flexibility in a measure of switching cost between congruent and incongruent trials in a similar task (i.e., they were slower than control peers, suggesting more difficulties in coping with incongruencies). While we did not identify a significant difference in switching cost in the current study, it is possible that the overall poorer performance in the MTT observed in girls at FHR for EDs reflects a similar dysfunction (Egner and Siqi‐Liu 2024; Braem and Egner 2018; Dreisbach and Fröber 2019). However, this interpretation requires careful consideration and further investigation. Of note, our results become more robust when older subjects are excluded, as this adjustment effectively eliminates age differences between the samples. It is plausible to hypothesise that the inclusion of participants across such a wide age range may have introduced variability that influenced the results of the analyses. Set‐shifting (a specific form of cognitive flexibility) has much evidence as a potential trait marker for AN (Malcolm and Phillipou 2021; Holliday et al. 2005), possibly contributing to the rigidity in thinking and behaviour experienced by individuals with AN in everyday life. Family members of ED patients (e.g. sisters) who do not suffer from ED also perform similarly to individuals with EDs (AN and BN) in set‐shifting (Tenconi et al. 2010; Roberts et al. 2010). In our exploratory analysis, girls of mothers with AN demonstrated the poorest performance in cognitive flexibility among the three ED subgroups. However, it is important to note that girls at FHR in all three subgroups exhibited worse cognitive flexibility compared to HC only in unadjusted analyses. Therefore, our findings regarding subgroup‐specific differences in maternal EDs should be interpreted with extreme caution. Our analyses were likely limited in statistical power, due to small sample size in individual EDs groups. More in‐depth studies with larger samples are needed to identify specific differences in maternal diagnosis. To sum up, given its presence in individuals at FHR for EDs at an early age, as well as in active patients (Brockmeyer et al. 2022; Miles, Gnatt, Phillipou, and Nedeljkovic 2020b) with the illness, and those in recovery (Miles, Gnatt, Phillipou, and Nedeljkovic 2020a), impaired cognitive flexibility seems a strong candidate as a trait marker of ED.

Conversely, we found no statistically significant differences in inhibitory control (impulse control) and working memory in girls at FHR for EDs compared to HC. Although some differences between groups emerged, these did not remain significant after statistical corrections. We speculate that the lack of observed differences may be attributed to factors such as the broad age range or presence of psychopathology. Another reason may be that we examined only one domain of inhibitory control, that is motor impulse control. Investigating inhibitory control more broadly, for example by examining the response to cognitive, emotional, and behavioural impulses might be necessary for future studies.

We did not find any differences in working memory. In particular, our data did not replicate the finding of a previous large‐scale study (Kothari et al. 2013) that showed that children of mothers with AN and BN had differences in working memory performance compared with control children (i.e. they were better than controls). Given that in our study we only investigated spatial working memory, this might again depend on the breadth of the different domains related to working memory studied, or the type of task used.

Girls at FHR for EDs showed structural differences in focal GM regions compared to HC girls. One of the clusters showing a statistically significant difference included the right supramarginal gyrus. There is not a well‐established consensus on the direct involvement of the right supramarginal gyrus in EDs. Situated within the parietal lobe, this region constitutes an integral component of the somatosensory association cortex. While it plays a role in diverse cognitive functions, there is a notable association between the right supramarginal gyrus and the regulation of empathy towards others (Silani et al. 2013; Schurz et al. 2017) and maintenance of emotion recognition ability (Wada et al. 2021). Patients with AN were found to have significantly lower cognitive empathy scores than controls (Kerr‐Gaffney et al. 2019), including perspective‐taking (Kerr‐Gaffney et al. 2019; Bora and Köse 2016), and theory of mind (Bora and Köse 2016). Differences within EDs were also reported, with individuals with AN showing poorer performance than those with BN and HC in an affective theory of mind task (Tapajóz Pereira de Sampaio et al. 2013). Further investigations either within a functional framework or investigating the relationship between structure and function, are needed to clarify whether this brain area may play a role in EDs.

Our result showed a significant cluster within the occipital lobe cortex, located at the level of the right secondary visual cortex and the right visual association cortex. The term “secondary visual cortex” is commonly used to refer to specific brain regions involved in visual processing that are recognized as components of the secondary visual pathway. Within the visual pathway of the brain, several regions work in a hierarchical fashion to process visual information. It is worth noting that these data are consistent with the results of our preliminary study (Pappaianni et al. 2023), in which we found aberrant functional connectivity in the medial visual network in children at FHR of ED compared to controls. Specifically, FHR children showed increased functional connectivity within the medial visual network, while in this case a higher concentration of GM was found in the same posterior regions. Interestingly, these results also included the right fusiform gyrus. The fusiform gyrus is a critical component of the visual system, helping to recognise and process complex visual stimuli, particularly faces and objects. However, the role of the fusiform gyrus in EDs is still very unclear. Previous studies, although inconsistent (Zhang et al. 2018) (Frank et al. 2013), have shown structural abnormalities in the fusiform gyrus in AN. From a functional point of view, individuals with AN show increased haemodynamic activity in bilateral fusiform gyri during the passive view of self‐images (McAdams et al. 2016), as well as during implicit emotion processing (Fonville et al. 2014). Moreover, brain activity alterations within the right fusiform gyrus were detected in individuals with AN and BN (Liu and et al. 2019) when compared to controls. The abnormal activation of the right fusiform gyrus in EDs was consistent across tasks domains, suggesting that this area may indeed play a key role in EDs. Therefore, abnormalities in the right fusiform gyrus may play a role in EDs psychopathology. Future studies should confirm this.

Lastly, girls at FHR for EDs showed increased CT compared to control girls in two cortical regions: the left transverse temporal gyrus and the right posterior cingulate cortex. The left transverse temporal gyrus is part of the primary auditory cortex, and it is responsible for processing auditory information. To our knowledge, there is no evidence specifically linking the neural abnormalities in the auditory cortex to EDs. The posterior cingulate cortex is involved in various cognitive and emotional tasks, and it is usually involved in cognitive control, emotional processing, self‐awareness. Involvement of the posterior cingulate cortex has been reported in active AN (Bär et al. 2015; Tose et al. 2024), with evidence of abnormal functional activation and lower cortical thickness and grey matter in this region. Moreover, individuals with BED and BN show abnormal activation of this area both during the expectation of food and food reward (Simon et al. 2016). We can speculate that neural abnormalities in the posterior cingulate cortex may be a biological trait marker of EDs or a reflection of developmental differences.

Our study identified differences in directionality of neural abnormalities between individuals in the acute phase (e.g., decreased CT and GMV) and children at FHR (e.g., increased CT). The aim of FHR studies is to tray and disentangle characteristics that might be illness trait markers (or endophenotypes) in individuals at high risk for a disorder but not presenting any active illness or related symptoms. Therefore, a difference in directionality of neural abnormalities maybe an important one and points to neural markers being a state marker of EDs rather than trait markers. A competing hypothesis is that children are still prone to neural pruning, especially at the level of CT. A third hypothesis is that given that FHR and HC girls differed in age, neural differences might be due to residual confounding.

Our study needs to be understood in the context of strengths and limitations. In terms of limitations, the groups differed in age. Unfortunately, the COVID‐19 pandemic had a major impact on our recruitment strategy, and we were unable to match our groups based on age. Acknowledging the potential influence of development on our results, we adjusted all analyses for age and further we excluded individuals with an age outside our chosen cut‐off for neurocognitive analyses, where age is likely to greatly impact on results due to cognitive development. In our brain analyses we constructed a customised tissue probability map for MRI images preprocessing, striving for optimal representation within our sample by considering both age and sex of the participants. Moreover, girls at FHR had a very high prevalence of psychiatric disorders other than ED. Although we adjusted all analyses to account for this variability, residual confounding cannot be excluded.

Second, we ran several linear regressions with different CANTAB outcomes as the dependent variable. Therefore, we cannot exclude the possibility of false positives given multiple testing.

In neural analyses, whilst we applied a correction for multiple comparisons in VBM (using FWE‐corrected level of significance), we did not apply any correction for the CT ROI analyses. Considering the limited sample size of the diagnostic subgroups, we opted against conducting neural analyses comparing each subgroup to mitigate the risk of interpreting results with insufficient statistical power.

Thirdly, we cannot exclude the possibility that the characteristics of our sample do not reflect those of the general population. Only 29% of the contacted mothers with lifetime history of EDs agreed to participate in the study, which could suggest a problem of sample representativeness. The main reasons for refusing to participate ranged from lack of time, to concern that their daughters might come into contact with EDs, to the memory of the painful experience undergone during treatment.

Finally, our sample was predominantly White European, possibly limiting the generalisability of our findings to other ethnicities.

## 5 Conclusions

In this FHR study we investigated neurocognitive function and brain structure in children at FHR of EDs compared to HC not at FHR for EDs. Corroborating previous preliminary evidence, our findings indicate a potential deficit in cognitive flexibility amongst girls at FHR of ED as trait marker of EDs. Additionally, we explored brain structure for the first time and identified differences in GM volume in posterior regions, potential trait markers or precursors of EDs. Specifically, regions such as the occipital cortex and the fusiform gyrus appear to be implicated. Future research endeavours should aim to validate our brain findings, employing longitudinal designs to elucidate whether these neural changes persist into adolescence and adulthood.

## Conflicts of Interest

The authors declare no conflicts of interest.

## Supporting information

Supporting Information S1

## Data Availability

The data that support the findings of this study are available from the corresponding author upon reasonable request.
