# Supplementary material for: Neurobiology and Cognition in Girls at High‐Risk of Eating Disorders: Exploring Imaging‐Derived Trait Markers
Source: Eur Eat Disord Rev. 2025 Apr 24;33(5):1032–43. doi: 10.1002/erv.3203 (PMC12319134; doi:10.1002/erv.3203)
Supplement: Supplementary file 1 — Supporting Information S1 [file ERV-33-1032-s001.docx]

# Supplementary materials

## SM1

### Recruitment process for patients and controls

Mothers with EDs were identified from three different sources.

1. The adult ED treatment centre at the Hôpitaux Universitaires de Genève (HUG): all women who were offered treatment who had children. A clinical register established by the unit was checked for potentially eligible women who had a diagnosis of AN, BN, or BED; 184 women with a lifetime history of EDs were identified via this source. Only these women were contacted by phone to determine if they had daughters in the inclusion age range, and, if so, they were asked if they wanted information about the study; amongst the 184 identified women with ED, 25 were unreachable via phone and 113 declined receiving information about the study/providing details about eligibility. Forty-six (46) women with lifetime history of EDs and a daughter in the inclusion age range agreed to receive information; 16 women decided not to take part in the study and 30 were eligible and agreed to participate.
2. Other private and canton-funded ED treatment centers: women under treatment for EDs at any of the collaborating treatment centers who had a daughter in the target age range were asked by their treatment provider if they were interested in participating in the study. In total, 16 eligible women with ED and daughters were identified via partnering centers. Only those who consented to be contacted by the research team were reached via phone/email (N= 10); Ten (10) women agreed to participate.
3. Advertisement (flyers and Social Media posts): 16 women contacted us after seeing adverts for the study.

Mothers without EDs were mainly recruited via advertisement, such as flyers delivered at the University of Geneva, at the HUG, and in local schools (N= 12); and posting on social media (Facebook, LinkedIn, Instagram, HUG website) (N= 30). Sixteen (16) control mothers were recruited via “word of mouth”.

All potentially eligible participants were contacted by the research team by email and/or telephone and provided information about the study, they were then screened by telephone to ascertain inclusion and exclusion criteria. Fifty-eight (58) mothers with EDs were eligible and gave informed consent to participate; Fifty-two (52) control mothers were considered eligible and gave informed consent to participate.

A recruitment flowchart is available for the group at-risk (S1-F1) and for HC (S1-F2).

SM1-F1. Girls at FHR for EDs - Flowchart of recruitment process and inclusion


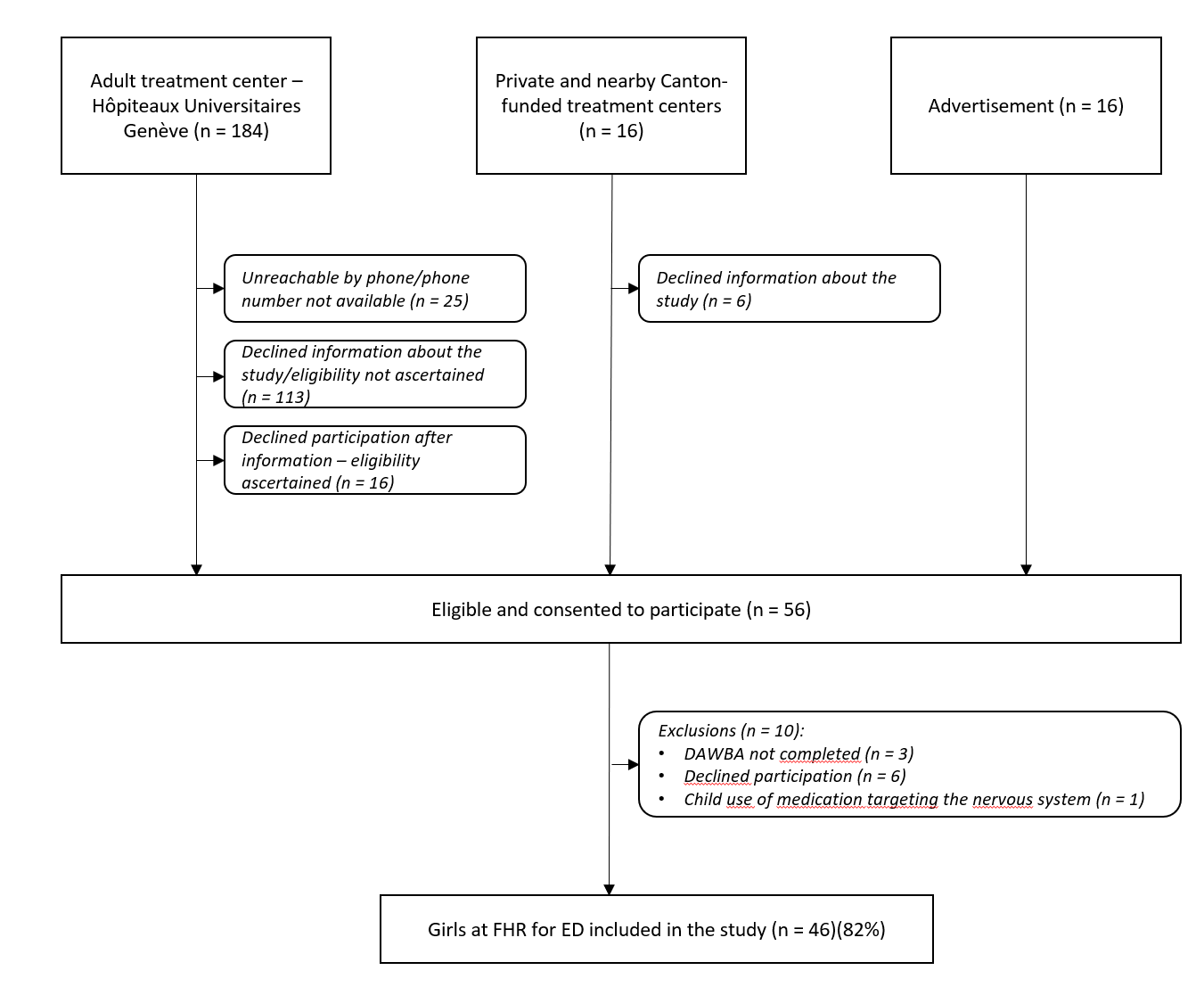


SM1-F2. Healthy controls - Flowchart of recruitment process and inclusion


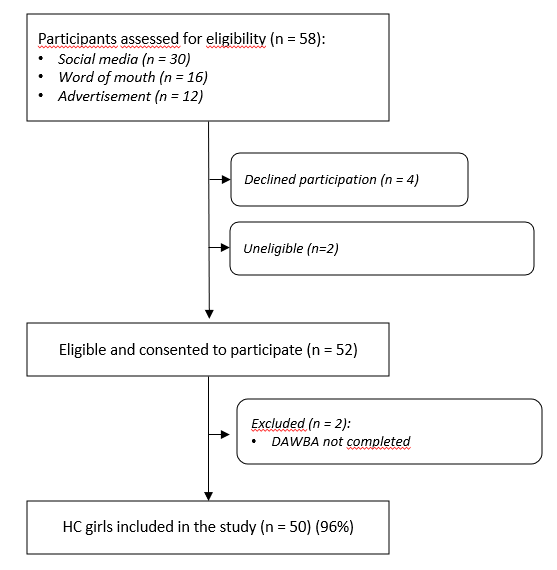


## SM2

### MRI procedure

Girls were scanned with a Siemens Prisma 3T scanner at Fondation Campus Biotech (Geneva, Switzerland). The complete imaging protocol included four main sequences: a structural T1-weighted Magnetization Prepared Rapid Acquisition Gradient Echo (MPRAGE) image, a task-based fMRI sequence, a diffusion-weighted (DWI) sequence and resting-state fMRI, with a total scanning duration of fortyfive minutes. For the purposes of this paper, only structural T1-weighted MPRAGE imaging will be considered. A 64-channel head coil was employed. The T1-weighted structural images were acquired with isotropic voxel resolution = 1.0 mm^3^, reaction time (TR) = 2200 ms, echo time (TE) = 2.96 ms, slice thickness = 1.0 mm, with an acquisition time of approximately 5 minutes. Girls were provided with a cartoon/movie of their choice, which was played on a screen that was installed at the bottom of the scanner and could be seen thanks to a system of mirrors that were placed above the coil.

## SM3

### Voxel-based Morphometry analysis

MRI images were checked for quality, abnormalities, motion and other artefacts. After careful quality control for artefacts, T1-weighted MPRAGE images were segmented into grey matter (GM), white matter (WM), and Cerebrospinal fluid (CSF). Given our unique population, an ad hoc tissue probability map template was created using the CerebroMatic toolbox^61^, based on the age, sex and magnetic field strength of our participants. GM, WM and CSF volumes, as well as Total Intracranial Volume (TIV) were extracted and quantified in all participants. GM, WM, CSF and TIV volumes were analysed using linear regression analysis with volumes as dependent variables and group, age and presence of psychiatric diagnosis (as assessed by DAWBA) as independent variables. Statistically significance level was set up at p < 0.05. GM images were then normalized to MNI space using the Diffeomorphic Anatomical Registration using Exponential Lie algebra (DARTEL) tools, a potential alternative to the traditional registration methods used by SPM. As last preprocessing step, spatial smoothing (using a full-width at half maximum Gaussian smoothing kernel from [8, 8, 8]), was then applied to the normalized GM images. VBM examines GM concentration at the voxel level and investigates differences between groups.
